# Editorial: Food bioactives: Cutting-edge methodologies for extraction and characterization

**DOI:** 10.3389/fnut.2022.1081974

**Published:** 2022-12-09

**Authors:** Jorge A. M. Pereira, Natalia Casado, José S. Câmara

**Affiliations:** ^1^CQM—Centro de Química da Madeira, Universidade da Madeira, Funchal, Portugal; ^2^Departamento de Tecnologia Química y Ambiental, Universidade da Madeira, Funchal, Portugal; ^3^Departamento de Química, Faculdade de Ciências Exatas e da Engenharia, Universidade da Madeira, Funchal, Portugal

**Keywords:** food composition analysis, dietary bioactive compounds, food samples extraction, microextraction, food analysis and composition

The knowledge about food composition, particularly concerning the different bioactive compounds it may contain, is very important at different levels of the food chain. It is necessary to help consumers make wiser and healthier dietary decisions and it is relevant to the farmers so they can select the most productive plants and manufacturing conditions as well as identify new or unexplored plants and food products. It is also crucial to the different food traders so they can offer the most appropriate solution for the target client and commercial environments, reflecting the costs of production, transformation, transport, storage, and marketing. Such knowledge, however, is not always readily accessible to the respective players. This may be due to several reasons, including the complexity of many food matrices, incomplete knowledge about the bioactivity of different food components or the cost of analysis, amongst others. In this context, the improvement of the analytical layouts necessary to characterize dietary bioactive compounds is very relevant.

This Research Topic aims to contribute to filling the gap in the knowledge about dietary bioactive compounds by presenting current and novel methodologies being used to obtain information about their presence, concentration, availability, and activity in different food products.

In this context, five papers were published in this Research Topic, two of which reviews devoted to microextraction in food analysis and assessment of anthocyanins in foods. The remaining contributions include original research about the bioactive composition of different foods. In the review, Pereira et al. unveiled the potential of microextraction techniques for the analysis of bioactive compounds in food. This focused review will give the reader an overview of the different microextractions approaches that researchers have already available to extract bioactive compounds from different foodstuffs, allowing a smooth migration from the traditional methodologies to innovative microextractions procedures. In the second review of this Research Topic, Manzoor et al. proposed a narrative review of the recent advances in the rapid assessment of anthocyanins in agricultural and food products. Due to their potential health benefits, anthocyanins are a very relevant group of bioactive compounds widespread in foods of vegetal origin. For this reason, their rapid assessment in foodstuffs is very important and so we believe the review proposed will be of interest to many readers. Regarding the original research, Xu et al. reported an integrated metabolomics approach to study a very popular tea among Chinese consumers which is obtained by a very peculiar process of fermenting Granpu tea inside a citrus fruit (Cachi). This cofermentation will favor the transfer of compounds from the citrus peel to the tea that was poorly characterized so far. Using an integrated metabolic approach involving UHPLC-QE Orbitrap MS-based qualitative and quantitative method combined with multivariate analysis, the authors were able to show a significative change in the chemical composition of pu-erh tea which includes the transfer of the bioactive hesperidin from the citrus peel to the tea. Also related to citrus fruits, Fan et al. proposed an optimized pulsed electric field-assisted extraction to enhance the yield and the physicochemical properties of soluble dietary fiber from orange peel. The study presented is of high relevance for the valorization of orange peels, given this is a waste produced in large amounts worldwide and their deposition in landfills has a high negative impact on the environment. Finally, Kalogiouri et al. proposed headspace solid-phase microextraction followed by gas chromatography-mass spectrometry to discriminate truffle species according to their volatile composition. Using this powerful analytical tool, the authors provided the first assessment of the volatile metabolome of the truffles *Tuber Aestivum* and *Tuber Borchii* originating from Greece. Truffles are fungi that grow below ground level, being highly appreciated for their nutritional value, health benefits, and unique organoleptic properties. For this reason, they are very expensive and so the fast and non-destructive sample extraction and analysis methodology proposed to discriminate truffles according to their origin has a high economic impact.

## Author contributions

All authors listed have made a substantial, direct, and intellectual contribution to the work and approved it for publication.

